# Physical and Sedentary Activity Patterns in Youths with Autism Spectrum Disorder

**DOI:** 10.3390/ijerph18041739

**Published:** 2021-02-11

**Authors:** Chien-Yu Pan, Chia-Liang Tsai, Fu-Chen Chen, Bik C. Chow, Chih-Chia Chen, Chia-Hua Chu

**Affiliations:** 1 Department of Physical Education, National Kaohsiung Normal University, No.116, He-Ping First Road, Kaohsiung 802, Taiwan; chpan@nknucc.nknu.edu.tw (C.-Y.P.); fcchen@nknu.edu.tw (F.-C.C.); 2 Institute of Physical Education, Health and Leisure Studies, National Cheng Kung University, Tainan 701, Taiwan; andytsai@mail.ncku.edu.tw; 3 Department of Sport, Physical Education and Health, Hong Kong Baptist University, Hong Kong, China; bchow@hkbu.edu.hk; 4 Department of Kinesiology, Mississippi State University, Starkville, MS 39762, USA; chih.chia.chen@msstate.edu

**Keywords:** accelerometer, health behavior, physical performance

## Abstract

Little is known about the patterns of sedentary behavior and physical activity (PA) within different school levels (i.e., primary school and secondary school) and on different day types (i.e., weekdays and weekend days) among youths with autism spectrum disorder (ASD). The sample was recruited from one city in Taiwan. A total of 68 male youths with ASD, aged 6–17 years, participated in the study. PA was assessed using an ActiGraph accelerometer, and sedentary behaviors (i.e., TV viewing, computer use, and reading time) were determined using a self-report log. The main findings were that (a) primary school youths with ASD were more active than secondary school youths with ASD on both weekdays and weekend days, but primary school youths with ASD also had more sedentary time than did secondary school youths with ASD on both weekdays and weekend days; (b) secondary school youths with ASD were more active but also more sedentary on weekdays compared with weekend days, but they had more screen use on weekend days compared with on weekdays. Future interventions are required to decrease sedentary behavior and increase PA to improve the health of these youths according to school level and day of the week.

## 1. Introduction

It is well understood that physical activity (PA) can provide many health benefits to children and adolescents (hereafter referred to as youths) who participate. The positive health outcomes for youths include enhanced weight control, maintained higher levels of cardiorespiratory fitness and stronger muscles, reduced risk of many chronic diseases [1], and improved core executive functions of memory, attention, and academic performance [2]. To achieve substantial health benefits, the 2018 PA Guidelines for Americans (second edition) recommend that school-aged youths (6–17 years) with and without disabilities participate in moderate-to-vigorous PA (MVPA) for up to 60 min or more each day [3]. Despite strong evidence that supports the importance of PA for health and quality of life, data obtained from across the world indicate that few youths with [4] and without disabilities [5,6] met this recommendation. Particular sub-groups among youths with a disability may have an especially high risk of inactivity, such as youths diagnosed as having autism spectrum disorder (ASD). Recent data indicate that among youths with various developmental disabilities, the proportion of those meeting these guidelines ranged from 14% to 20%; youths with ASD had the lowest proportion [4].

ASD is clinically defined by impairments in social and communication abilities associated with symptoms varying from difficulty in communication and social interaction to restricted and repetitive behaviors [7]. Further, individuals with ASD may exhibit delayed motor development [8] and a lack of motivation to engage in PA [9]. Parents and clinicians have reported that social, cognitive, behavioral, and physical limitations experienced by individuals with ASD tend to prevent them from participating in PA [10]. Despite these barriers, research has shown that PA reduces typical autistic symptoms [11,12] and daily emotional and behavioral problems [12] and promotes sensory skills and social responsiveness [13] as well as motor skill proficiency and executive function [14] among youths with ASD. Therefore, similar to typically developing (TD) youths [9], youths with ASD could benefit from lifestyle modifications that promote increased PA and reduce negative behaviors.

The literature reporting objectively measured PA levels in youths with ASD is sparse; most studies have been limited to various age groups [10,15] or restricted to a particular part of the day only (e.g., physical education class, recess, after school) [16,17]. A systematic review by Jones et al. [18] included 13 studies using objective measures of PA and reported that average MVPA ranged from 34 to 165 min/day (average 90 min/day), and average total PA ranged from 361 to 1763 counts/min (CPM, mean 826 CPM). Data reported from accelerometers were inconsistent. For example, Bandini et al. [10] compared accelerometry PA data in youths with and without ASD aged 3–11 years. They found that both groups of youths engaged in similar levels of MVPA, as measured by accelerometry over 1 week (seven consecutive days; ASD: 50.0 min/day; TD: 57.1 min/day), during weekdays (ASD: 48.0 min/day; TD: 59.2 min/day), and during weekend days (ASD: 53.5 min/day; TD: 52.1 min/day). However, in a secondary analysis, after age and gender were controlled for, it was revealed that time spent in moderate PA (MPA) on weekdays was higher in TD youths (57.6 min/day) than did youths with ASD (46.5 min/day). Percentage of the youths with ASD accumulated for at least 60 min daily MVPA was only 23%. Rosser-Sandt and Frey [17] indicated that there were no differences between youths with ASD and TD youths aged 5–12 years, in daily PA levels and at any PA setting (physical education class, recess, and after school). There were no weekday and weekend day MVPA differences between youths with ASD and TD youths, and 67% of the youths with ASD achieve daily 60 min of MVPA recommendations. Memari et al. [19] investigated PA patterns in youths with ASD aged 7–14 years and found that the level of PA of youths with ASD was significantly less in school than that after school, and the difference between the level of PA during weekdays and weekend days was not statistically significant. Tatsumi et al. [20] reported that pre-schoolers with ASD had significantly lower CPM (630.5) on weekend day mornings compared with TD youths (746.0); CPM did not vary for other times of the week or other periods of weekend days (afternoon or evening). By contrast, other studies have suggested that youths with ASD do not demonstrate PA deficits relative to their same-age peers. For example, Ketcheson et al. [21] revealed that youths with ASD aged 2–5 years spent significantly more time in accelerometer-measured MVPA compared with youths without ASD. Pan and Frey [22] investigated PA patterns in youths with ASD aged 10–19 years and observed no differences in overall PA or MVPA regarding day-of-week and time-of-day variability according to participants’ school level. MacDonald, Esposito, and Ulrich [23] assessed the MVPA of 72 youths with ASD aged 9–18 years and determined that both the younger (9–11 years) and older (12–18 years) youths met the minimal recommendation of 60 min of daily MVPA. Collectively, these studies highlight that the extent to which youths with ASD engage in sufficient amounts of PA remains unclear and suggest a need to investigate the potential reasons for this inconsistency.

Emerging evidence indicates that sedentary behavior has a notable negative influence on various health indicators [24]. “Sedentary behavior is any waking behavior characterized by an energy expenditure less than or equal to 1.5 metabolic equivalents, while in a sitting, reclining or lying posture” ([3], p. 21). Common sedentary behaviors include television (TV) viewing, video game playing, computer use (collective termed “screen time”), driving automobiles, and reading. The American Academy of Pediatrics recommends that youths, aged 6 years and older, have 2 h or less of sedentary screen time daily [25]. However, current evidence indicates that among youths with ASD, subjectively measured total screen time (e.g., TV viewing, video-gaming, and device use) ranged from 86 to 430 min/day (average 231 min/day), and objectively measured total sedentary time ranged from 312 to 789 min/day (average 514 min/day) [18]. Despite the substantial variations in estimates of screen time, few studies have revealed that youths with ASD spend more time engaging in sedentary activity than do TD youths [26,27,28], although this finding has not been consistently observed across all age groups [29] and varies based on method of data collection (e.g., survey [26,29] or accelerometry [21,27,28]). Chonchaiya et al. [26] found that youths with ASD had significantly more screen time than TD youths (4.6 vs. 2.6 h/day) and fewer complied with screen time recommendations of less than 2 h/day (6% vs. 44%) compared with their TD counterparts. Lobenius-Palmér et al. [27] indicated that youths with ASD (aged 7–20 years) had more sedentary time than TD youths (458 vs. 350 min/day). Youths with ASD (aged 9–17 years) in Tylers’ [28] study were also more sedentary than TD youths (452 vs. 369 min/day). By contrast, Ketcheson et al. [21] found that youths with ASD (aged 2–5 years) spent significantly less time per day engaging in sedentary activity compared with the TD group (577 vs. 655 min/day). Dreyer Gillette et al. [29] found that a significantly higher percentage of youths with ASD (19.8%) never used a computer, cell-phone, hand-held-video-games, or other electronic devices compared with their TD counterparts (8.7%). However, their study also found no difference in youths’ use of TV, video, and video-games, or in the number of hours of use of computers, cell-phones, hand-held video-games, or other electronic devices. Nevertheless, the prevalence rate of sedentary activities among youths with ASD largely remains unknown.

To design interventions for youths with ASD, it is first necessary to determine levels of physical and sedentary activity and how physical and sedentary activity are influenced by different factors, such as school level (e.g., primary school or secondary school) and day of the week (e.g., school days/weekdays or non-school days/weekend days). At different school levels and day types, youths spend time in different social (e.g., students and family) and organizational environments that influence levels of physical and sedentary activity. However, few accelerometer studies have addressed these factors in youths with ASD, limiting knowledge of the impact of school level and day of the week on patterns of physical and sedentary activity. Must et al. [30] found that youths with ASD aged 3–11 years had longer average weekday and weekend day TV viewing times and total screen times (TV, video-game, and computer) than did TD youths, although TV viewing time and total screen time was longer on weekend days compared with weekdays for both groups. Furthermore, a significant and positive correlation was observed between age and weekend total screen time among youths with ASD. In a previous publication based on a systematic review of 35 studies among individuals with ASD aged 2–21 years old, age was consistently inversely associated with PA, and age was inconsistently associated with sedentary behaviors [18]. Individuals with ASD often have repetitive interests and activities in many areas of everyday life; therefore, the day of the week plays a critical role in terms of them planning and organizing their behaviors. Thus, examining day-of-week physical and sedentary activity patterns among youths with ASD can be informative and helpful for designing future interventions. To date, no clear evidence is available on the prevalence of physical and sedentary activities in a wide range of school-age youths with ASD on both weekdays and weekend days.

The education system in Taiwan is made up of up to 12 years of compulsory schooling: 6 years of elementary (primary) school (grades 1 to 6), 3 years of junior high school (grades 7 to 9), and 3 years of senior high school or vocational education (grades 10 to 12). Students’ PA levels are particularly crucial because the majority of Taiwanese families live in small apartments in high-rise buildings, and space for PA in schools and communities is limited. Because previous studies have been conducted in the West, this study may increase our understanding of physical and sedentary activity patterns in the population with ASD across cultures. Therefore, the aim of this study was to compare physical and sedentary activity patterns of Taiwanese youths with ASD at different school levels (i.e., primary school and secondary school) and on different day types (i.e., weekdays and weekend days). We hypothesized that physical and sedentary activity patterns in this population may differ by school levels and that these differences would be evident across weekdays and weekend days.

## 2. Method

### 2.1. Participants

Youths with ASD between the ages of 6 and 17 years were recruited for this study. In an effort to obtain a homogenous sample, participants were recruited in the same geographical area of a large urban city in Taiwan with high social and economic deprivation. Recruitment was conducted at local schools and local rehabilitation facilities as well as through autism-based programs, the dissemination of study flyers, and word of mouth. Only male youths were included in the study because of gender ratio disparities in ASD (approximately 4:1 male: female) [31]. All participants with ASD had been diagnosed when they were toddlers and received a new clinical diagnosis according to the Diagnostic and Statistical Manual of Mental Disorders, Fourth Edition, Text Revision [32] criteria for ASD from a child psychiatrist or hospital physician [33] within 2 years before the present study began. We recruited 68 participants with a clinical diagnosis either of autistic disorder (*n* = 47) or Asperger syndrome (*n* = 21). Each diagnosis was identified on the participant’s disability card as issued by the local government. In addition, the clinical diagnoses of autistic disorder and Asperger syndrome made by the psychiatrist or physician were further confirmed using the traditional Mandarin version of the Autism Behavior Checklist [34] and Autism/Asperger Behavior Rating Scale [35] to screen for autism and Asperger behaviors, based on parent ratings. None of the participants was identified to have a co-occurring intellectual disability based on the diagnosis statement from the psychiatrist or the physician. All participants were in good health and free from diseases or disorders that could affect PA habits (e.g., diabetes, cerebral palsy, orthopedic dysfunction, etc.).

### 2.2. Procedures

The study was conducted after approval by the Human Research Ethics Committee (ID: 106-026) of National Cheng Kung University. Written parental consent and youth assent were obtained for all participants after a detailed explanation of the objective and procedures of the study. All participants were weighed and measured in light clothing without shoes in a private setting. Body mass index (BMI) was calculated from measures of height and weight (kg/m^2^) and then transformed to an age- and sex-specific z-score.

### 2.3. Accelerometer-Assessed PA and Sedentary Time

Participating youths wore a GT1M ActiGraph accelerometer (Pensacola, FL, USA), an objective measure of PA and sedentary time widely used for youths with and without disabilities. Research examining this accelerometer suggests that it is a valid and reliable measurement of PA and sedentary time over a seven day period for youths with disabilities [36]. Youths with ASD were found that they can tolerate using accelerometers [22]. All participants were asked to wear the accelerometer for no less than 10 h a day during data collection. They were asked to wear the accelerometer on the right hip using an elastic belt, for seven consecutive days, except while sleeping, showering, bathing, or during other aquatic activities. A self-report log was provided to record when the accelerometer was worn and removed.

Outcome variables were total PA (CPM) and time spent (min/day) in sedentary, light PA (LPA), and MVPA, and these were derived from established cut-off points [37]. These cut-off points (sedentary: ≤100 CPM; LPA: 101–2295 CPM; MVPA: ≥2296 CPM) were recommended to estimate time spent in different PA intensity in children and adolescents [38]. At the same time, accelerometer data were divided into weekdays (Monday-Friday) and weekend days (Saturday and Sunday). Data were downloaded using the ActiLife 6 Software (v.6.12.0., ActiGraph Pensacola, FL, USA) and reduced into average time spent in the PA categories described. Zero activity counts lasting longer than 60 min were considered a non-wear period and therefore excluded from data analysis.

### 2.4. Self-Reported Sedentary Behavior

Alongside PA, three different sedentary behaviors (TV viewing, computer use, and reading time) were assessed using a self-report log. All participants were asked to take a packet of logs home. This self-report log was evaluated each day during the same period the accelerometer was worn. Each sedentary behavior was measured by asking youths the total number of minutes per day spent doing each activity that day (all day for the weekend days and only after school (4:00 p.m. to bedtime) during weekdays). Parents/caregivers can assist their children to recall the activities they participated in each day and circle the total number of minutes of participation for each activity that day.

### 2.5. Statistical Analysis

The mean (and standard deviation (SD)) for each variable was calculated for participants with ASD. For inclusion in the PA analyses, participants were required to wear the accelerometer for a minimum of 8 h each day for at least four weekdays and one weekend day [36].

The nonparametric Mann–Whitney U test was used to examine significant differences in the means of the weekly PA and sedentary behaviors between primary and secondary school participants with ASD. Next, because of the small sample size and unbalanced groups, the nonparametric Mann–Whitney U test and the nonparametric Wilcoxon signed-rank test were used to compare PA and sedentary behaviors between and within the two school-level groups on weekdays and weekend days. Secondary analyses were conducted using chi-square tests to compare the percentage of fulfillment of the daily 60-min MVPA recommendation and rate of compliance with screen media guidelines between primary and secondary school youths with ASD. All statistical analyses were performed using SPSS version 20 (SPSS Inc., Chicago, IL, USA). The significance level was set at *p* < 0.05.

## 3. Results

The characteristics of all participants are presented in Table 1. Sixty-eight youths with ASD were recruited in the study, and all youths with ASD completed the study. Regarding accelerometry, data from 39 days were excluded, from a maximal potential monitoring period of 476 days, because of insufficient wear time (<8 h).

### 3.1. Meeting PA Recommendations

Table 1 shows the proportion of the participants who met the daily 60-min MVPA recommendation. For all participants with ASD, 69% (*n* = 47) accumulated at least 60 min of daily MVPA during seven monitored days. Regarding the day of the week, 72% (*n* = 49) and 52% (*n* = 35) satisfied the recommended MVPA on weekdays and weekend days, respectively.

Fewer secondary-school-aged youths with ASD met the recommended levels than did primary-school-aged youths with ASD throughout the week (χ^2^ = 34.18, *p* < 0.01), and both on weekdays (χ^2^ = 29.66, *p* < 0.01) and on weekend days (χ^2^ = 16.96, *p* < 0.01).

### 3.2. Rate of Fulfillment of Screen Media Guidelines

Descriptive statistics for screen media behaviors (i.e., TV viewing and computer use) for weekdays and weekend days by school levels are presented in Table 1. Of all participants with ASD, 41% (*n* = 28), 54% (*n* = 37) and 37% (*n* = 24) fulfilled the screen media recommendation during the seven monitored days, on weekdays, and on weekend days, respectively.

The results of chi-square tests indicated that the percentages of compliance with screen media guidelines between primary and secondary school youths with ASD were not significantly different throughout the week (χ^2^ = 3.61, *p* = 0.06), on weekdays (χ^2^ = 2.46, *p* = 0.12), and on weekend days (χ^2^ = 0.81, *p* = 0.37).

### 3.3. Patterns of Physical and Sedentary Activity Throughout the Week

The 68 participants had an average (SD) monitoring period of 6.43 (0.70) days. The minimum requirements for valid accelerometer data were 8 h per day. All participants in the present study provided at least five days (including one weekend day) of completed monitoring.

Table 2 presents the mean minutes of intensity-specific physical and sedentary activity patterns over a seven day period. We observed no differences in the total monitored length between school levels (Z = −1.73, *p* = 0.08). The rates of compliance with screen media guidelines (less than 2 h per day) are also displayed in Table 2 relative to these variables.

The statistical analyses of the differences between school levels in relation to each PA intensity level revealed that secondary school youths with ASD spent significantly less time engaging in MVPA (Z = −5.91, *p* < 0.05) and had significantly higher CPM (Z = −4.29, *p* < 0.05) within a seven day period compared with primary school youths with ASD, and secondary school youths with ASD spent significantly more time engaging in LPA (Z = −7.08, *p* < 0.05) throughout the week than did their primary school counterparts. Regarding sedentary time and sedentary behavior outcomes, the results showed that, over a seven day period, primary school youths with ASD had significantly more sedentary time (Z = −4.20, *p* < 0.05) and TV viewing time (Z = −2.95, *p* < 0.05) than secondary school youths with ASD.

### 3.4. Patterns of Physical and Sedentary Activity by School Level and Day Type

Table 3 presents physical and sedentary activity outcomes during weekdays and weekend days by school level and day type. We observed no school-level (weekdays: Z = −1.22, *p* = 0.22; weekend days: Z = −0.05, *p* = 0.96) or day-type (primary school: Z = −1.05, *p* = 0.29; secondary school: Z = −1.05, *p* = 0.05) differences in the total monitored length.

On weekdays, compared with secondary school youths with ASD, primary school youths with ASD spent significantly more time engaging in daily MVPA (Z = −5.87, *p* < 0.05) and had significantly higher CPM (Z = −3.35, *p* < 0.05). Primary school youths with ASD also had more sedentary time (Z = −2.09, *p* < 0.05) and less LPA (Z = −7.08, *p* < 0.05). Primary school youths with ASD spent significantly more time on TV viewing (Z = −3.79, *p* < 0.05) than did secondary school youths with ASD, and secondary school youths with ASD spent significantly more time reading (Z = −2.19, *p* < 0.05). During weekend days, primary school youths with ASD had significantly longer sedentary time (Z = −3.85, *p* < 0.05) and time spent engaging in daily MVPA (Z = −4.98, *p* < 0.05) as well as significantly higher CPM (Z = −3.98, *p* < 0.05). No differences were found between primary and secondary school youths with ASD in any sedentary behavior.

In addition, the PA differences among the days of the week within each school-level group revealed that in secondary school youths with ASD, sedentary time (Z = −2.32, *p* < 0.05) was longer and MVPA (Z = −3.24, *p* < 0.05) and CPM (Z = −2.36, *p* < 0.05) were all higher during weekdays than during weekend days. Weekday reading time (Z = −2.36, *p* < 0.05) was significantly longer than on weekend days, whereas weekend day computer use (Z = −3.59, *p* < 0.05) and TV viewing (Z = −2.96, *p* < 0.05) times were significantly longer than on weekdays. For primary school youths with ASD, computer use (Z = −3.51, *p* < 0.05) and reading (Z = −3.87, *p* < 0.05) times on weekend days were both significantly longer than on weekdays.

## 4. Discussion

Our study adds to the few published studies that characterize patterns of physical and sedentary activities, specifically in youths with ASD and in separate weekdays/weekend days and primary/secondary school contexts. Based on a small sample size, the main findings of this study are that (a) primary school youths with ASD were more active (i.e., performed more MVPA and had higher a CPM) than secondary school youths with ASD on weekdays and weekend days, but primary school youths with ASD also had more sedentary time than secondary school youths with ASD on weekdays and weekend days; (b) secondary school youths with ASD engaged in more LPA than primary school youths with ASD on weekdays and weekend days; and (c) primary school youths with ASD spent more screen media time (i.e., TV viewing and computer use) on weekdays as compared with secondary school youths with ASD. In addition, secondary school youths with ASD performed more MVPA, had higher CPM, and had longer sedentary time on weekdays compared with weekend days, and they engaged in more screen media use on weekend days compared with weekdays. These results indicate that primary and secondary school youths with ASD should engage in activities specific to school level (i.e., primary and secondary school) and day of the week to increase their PA levels and decrease time spent using screen media.

In the present study, the MVPA of 69% of youths with ASD satisfied the World Health Organization (WHO) PA guideline. The value found for youths with ASD was higher than those found by Case et al. [4] (14%) and by Bandini et al. [10] (23%) and was similar to the values obtained by Rosser-Sandt and Frey [17] (67%) but lower than the values found by MacDonald et al. [23] (100%). When presented by school level, 100% of primary school youths with ASD and 34% of secondary school youths with ASD satisfied the recommendation. Worryingly, in our study, the percentage of secondary school youths with ASD satisfying the WHO recommendations was remarkably low; nevertheless, this percentage was slightly higher than that reported in the review by Jones et al. [18] for youths with ASD (ranging from 21% to 100%) and higher than that of Swedish male TD youths aged 7–20 years (less than 29% engaged in the recommended PA levels) [27]. These mixed findings that youths with ASD have somewhat different PA levels lead us to speculate if such findings might be partly attributable to the social model of disability [39], which recommends that societies provide appropriate services to ensure that the needs of individuals with ASD are fulfilled. Sample and cultural differences, among other personal and environmental factors, may also account for actual differences in time spent engaging in MVPA. This topic is not fully explored in this population and requires further investigation.

The average time engaging in MVPA in our study population (~81 min per day) was longer than that found for youths with ASD by Bandini et al. [10] (~50 min per day) and Ayvazoglu et al. [15] (~34 min per day), similar to that obtained by Obrusnikova and Cavalier [40] (~82 min per day), and lower than that those determined by Rosser-Sandt and Frey [17] (~128 min per day) and Tyler et al. [28] (~166 min per day). However, when the participants were divided by school level, primary school youths with ASD averaged approximately 104 min per day, and secondary school youths with ASD averaged approximately 56 min per day. These values were lower than those obtained by MacDonald et al. [23] for youths with ASD aged 9–11 years (~132 min per day) and 12–18 years (~90 min per day) and the values obtained in another study [22] for a group of elementary-school (~132 min per day) and middle-school (~75 min per day) youths with ASD, but they were higher than those obtained for high school youths with ASD (~40 min per day). These differences between results could be due to the different mean ages of their groups. However, the results of this study clearly show declines in PA as youths with ASD age. Several factors may make children more sedentary in their transition to adolescence. First, as youths get older, physical activities become more complex and competitive, and thus youths with ASD may have trouble following rules and accomplishing complex motor tasks. Moreover, the opportunities for participating in unstructured activities that constitute a major part of their PA, such as play games, transportation, chores, and recreation, will be partially missed [41]. Finally, youths with ASD are more likely to avoid participation in PA because of a lack of positive and rewarding experiences in play and physical activities [19]. This can foster feelings of failure and lead to low self-esteem as well as a lack of competency in physical and sport situations [42,43]. The observed age-related declines provide insight into the lack of PA demonstrated by secondary school youths with ASD. Nevertheless, it is important to note the volume of LPA among secondary school youths with ASD may have critical health implications because research indicated that LPA has significant acute and long term benefits for adult cardiometabolic health and mortality risk [44]. LPA may be particularly appropriate in contexts such as secondary schools where participation in formal PA programs and MVPA is typically low in youths with ASD. These results highlight a need for extracurricular after school programs for secondary school youths with ASD.

After splitting our data by day of the week, we found that the MVPA values for secondary school youths with ASD were lower than those obtained for primary school youths with ASD on weekdays and weekend days, and the weekend days’ MVPA values were lower than those for weekdays for secondary school youths with ASD. These MVPA values for secondary school youths with ASD were lower than those reported by Wachob and Lorenzi [45] (weekdays: 76 min per day; weekend days: 63 min per day) and were similar for primary school youths with ASD, as reported by Pan et al. [46] (weekdays: 104 min per day; weekend days: 99 min per day). In line with another study [47], we found no differences in MVPA between days of the week for primary school youths with ASD. These differences between studies may be attributable to the age-related decrease in PA [23] or to a lack of motivation in secondary school youths with ASD [9]. This could also be due to low self-perceived social competence [42] and low self-perception of physical condition, and poor motor skill competence [43] because research has indicated that secondary school youths with ASD had increased awareness of their own social deficiencies and poor competence in physical domains, leading to a decrease in PA participation. In addition to the youth-level barriers, Pan, Tsai, and Hsieh [9] examined PA correlates for youths with ASD during secondary school physical education class and found that PA during school physical education was positively associated with their social interactions with their peers, and MVPA was depended on PA content (i.e., team activities, individual activities, fitness testing, and free play), physical environment (i.e., indoor, outdoor, or a combination) and instructor-related characteristics (i.e., gender and major). The availability of physical and community resources may help to increase participation in PA in this population. School policies toward inclusion may provide students with disabilities and other marginalized groups (e.g., international student-athletes, LGBTQ (lesbian, gay, bisexual, transgender, queer) people, racialized people, and women) [47] equal opportunities to participate in PA [48].

The effect of the day of the week on sedentary activities was also more obvious in secondary school youths with ASD than in their primary school counterparts. In this study, we found less compliance with screen media use recommendations on weekend days (TV viewing: 83 min per day; computer use: 71 min per day) than on weekdays (TV viewing: 51 min per day; computer use: 32 min per day) for secondary school youths with ASD, and no day-of-week differences were found on TV viewing time for primary school youths with ASD, although such youths still exceeded 2 h of screen media per day on both weekdays (TV viewing: 128 min per day; computer use: 15 min per day) and weekend days (TV viewing: 111 min per day; computer use: 55 min per day). TV viewing and computer use were the screen media behaviors that youths engaged in for longer on weekend days, consistent with other studies [49,50,51]. The high engagement in screen media behaviors found in the current study for youths with ASD may be because that all participants resided in the same geographical area of a large urban city in Taiwan with high social and economic deprivation, and all attended public schools. Neighborhood and type of school are important factors associated with sedentary behaviors in ecological approaches [52]. Urban residence has been linked to more time spent engaging in sedentary behaviors in some studies [53] but not others [54]. Public secondary school students spend more time on screen media because higher participation in organized activities is often not economically feasible for these students [55,56]. Furthermore, participants reported different levels of each sedentary behavior, depending on the day of the week: computer use and reading times were longer on weekend days for both primary and secondary school youths with ASD, and TV viewing time was longer on weekend days for secondary school youths with ASD. Therefore, the day of the week may be related to the organization of leisure time for these youths. On weekend days, secondary school aged youths have more autonomy and free time and can manage their own time without compulsory activities; thus, they spend more time on screen media use. Behaviors on weekend days may be more varied than during weekdays because of reduced parental control [49] and supervision [56]. However, this result leads us to suggest that screen media use and PA may not be simply opposed to one another because youths can be physically active and still not meet screen media recommendations [57].

The high level of screen media usage and high adherence to MVPA recommendations found in primary school youths with ASD are notable. This finding is in concordance with the results reported by Melkevik et al. [58] and did not support the “displacement hypothesis” proposing that screen media use displaces PA [59]. The commonly observed decrease in PA in youths [60] had been found to be related to an increase in sedentary behaviors [61]. However, only slight associations were found in the meta-analysis of Biddle et al. [62]. Other studies (e.g., [63]) did not suggest that these behaviors directly displace one another. Indeed, physical inactivity is more complex than we sometimes think. Sedentary behaviors are largely uncorrelated with PA, suggesting that there is time for both [62] and they be viewed as separate constructs [64].

## 5. Limitations and Conclusions

The study had several limitations. First, it was a cross-sectional study; thus, attributions of causality are not possible. Second, the small sample size and no power analysis was performed on the sample, and therefore generalization of the current findings should be performed with caution. Third, ActiGraph accelerometers may have underestimated real PA levels because they were mounted at the hip level, and PA or movements with the upper body were not measured. Also, these accelerometers cannot be worn during swimming, bathing, or cycling activities. Fourth, the accelerometers could not distinguish between types of activity or body postures. For example, a distinction between standing and sitting may be essential, and yet periods of standing would be classified as sedentary time or LPA due to low acceleration. Fifth, although cut points were recommended [38], it could still over or underestimate true PA levels of participants in this study. In addition, accelerometers and their corresponding cut-offs for levels of PA have not been validated for youths with ASD. Because there is no consensus on which cut points to use for either for youths with ASD or TD youths, different cut-offs used and different cultural contexts make it difficult to directly compare across studies. Sixth, self-reported sedentary behavior in individuals with ASD may be open to recall errors; using device-based measures to determine sedentary behavior in future studies should be considered. Also, the lack of a control group is needed to be addressed as a limitation. Finally, it is necessary to recognize the problem of a lack of standardization of sedentary behavior measurements, making clear comparisons between studies complicated.

One of the primary strengths of the study was the assessment of PA using an objective measure in a population which may not get enough attention. In addition, this study does provide data on different school levels (i.e., primary vs. secondary school) and different day types (i.e., weekdays vs. weekend days) to better understand PA in youths with ASD. This information provides more areas for possible intervention strategies to overcome the barriers of PA in youths with ASD.

In conclusion, the results from the present study show that youths with ASD have lower PA levels and higher screen and sedentary time as children mature into adolescence. The majority of primary school youths with ASD exceeded the screen media use guidelines but were physically active throughout the week, during weekdays, and on weekend days. The majority of secondary school youths with ASD did not meet the PA recommendations and spent more time on screen media use on weekend days. These findings suggest that it may be important to implement different strategies to decrease screen media use and to increase PA to improve the health of these youths according to school level and the day of the week.

## Figures and Tables

**Table 1:** Characteristics of youths with autism spectrum disorder (ASD).

Variables	All *n* = 68	Primary *n* = 36	Secondary *n* = 32
Age (years)	11.74 ± 3.07	9.35 ± 1.79	14.42 ± 1.62
Height (cm)	150.51 ± 18.08	136.00 ± 10.76	166.84 ± 7.51
Weight (kg)	47.69 ± 17.67	34.95 ± 9.42	62.03 ± 13.20
BMI (kg/m^2^)	20.37 ± 4.79	18.68 ± 3.57	22.27 ± 5.31
BMI z-score	0.39 ± 1.19	0.34 ± 1.15	0.45 ± 1.26
Meeting PA guideline (*n*, %) ^a^			
Weekdays	49 (72%)	36 (100%)	13 (41%)
Weekend days	35 (52%)	27 (75%)	8 (25%)
All week	47 (69%)	36 (100%)	11 (34%)
Meeting screen media guideline (*n*, %) ^b^			
Weekdays	40 (59%)	18 (50%)	22 (69%)
Weekend days	28 (41%)	13 (36%)	15 (47%)
All week	30 (44%)	12 (33%)	18 (56%)

Mean ± standard deviation (SD); ^a^ ≧60 min moderate-to-vigorous physical activity (MVPA) daily measured using the ActiGraph accelerometer; ^b^ ≤120 min screen media use (i.e., TV viewing and computer use) daily assessed using a self-report diary.

**Table 2:** Patterns of sedentary behavior and physical activity (minutes/day) throughout the week in youths with autistic spectrum disorder (ASD).

Variables	All *n* = 68	Primary *n* = 36	Secondary *n* = 32	*p*-Value
**Subjectively measured sedentary behavior**				
TV viewing	94.68 ± 73.99	119.42 ± 73.02	66.84 ± 65.59	**0.003**
Computer use	42.72 ± 51.11	34.83 ± 41.74	51.60 ± 59.37	0.452
Reading	85.61 ± 68.74	79.75 ± 56.57	92.21 ± 80.71	0.796
**Objectively measured sedentary behavior**				
Wearing time	807.96 ± 65.02	795.94 ± 48.86	821.50 ± 78.00	0.083
Sedentary time	650.60 ± 80.74	689.82 ± 54.08	606.47 ± 83.61	**0.000**
**Objectively measured physical activity**				
LPA	75.31 ± 84.53	0.85 ± 0.15	159.09 ± 41.92	**0.000**
MVPA	81.44 ± 34.86	104.10 ± 29.01	55.94 ± 20.23	**0.000**
CPM	354.99 ± 115.03	405.84 ± 88.44	297.79 ± 115.71	**0.000**

Statistically significant values are shown in bold (*p* < 0.05). LPA: light physical activity. MVPA: moderate-to-vigorous physical activity. CPM: counts per minute.

**Table 3:** Patterns of sedentary behavior and physical activity (minutes/day) between primary and secondary school youths with autistic spectrum disorder (ASD) on weekdays and weekends.

Variables	Weekday		*p*-Value	Weekend		*p*-Value
	Primary	Secondary		Primary	Secondary	
**Subjectively measured sedentary behavior**						
TV viewing	128.00 ± 83.88	50.72 ± 56.71 ^b^	**0.000**	110.83 ± 83.58	82.97 ± 84.16	0.124
Computer use	14.67 ± 23.67 ^a^	31.95 ± 43.81 ^b^	0.220	55.00 ± 70.45	71.25 ± 81.89	0.473
Reading	55.33 ± 43.25 ^a^	108.02 ± 92.68 ^b^	**0.029**	104.17 ± 79.69	76.41 ± 86.91	0.153
**Objectively measured sedentary behavior**						
Wearing time	813.00 ± 61.85	827.87 ± 98.32	0.224	791.00 ± 149.54	785.44 ± 86.45	0.961
Sedentary time	684.30 ± 129.00	616.13 ± 98.14 ^b^	**0.037**	688.48 ± 133.32	564.19 ± 83.59	**0.000**
**Objectively measured physical activity**						
LPA	0.85 ± 0.16	153.21 ± 43.72	**0.000**	0.81 ± 0.22	175.38 ± 61.32	**0.000**
MVPA	104.27 ± 25.74	58.84 ± 22.21 ^b^	**0.000**	101.71 ± 49.16	45.86 ± 23.08	**0.000**
CPM	402.22 ± 93.50	381.31 ± 434.09 ^b^	**0.001**	414.24 ± 151.31	268.11 ± 118.92	**0.000**

Statistically significant values are shown in bold (*p* < 0.05). ^a^ Significant differences between day types among primary school youths with ASD (*p* < 0.05). ^b^ Significant differences between day types among secondary school youths with ASD (*p* < 0.05). LPA: light physical activity. MVPA: moderate-to-vigorous physical activity. CPM: counts per minute.

## Data Availability

Not applicable.
